# Slower Auger Recombination in 12-Faceted Dodecahedron CsPbBr_3_ Nanocrystals

**DOI:** 10.1021/acs.jpclett.2c03389

**Published:** 2023-01-25

**Authors:** Supriya Ghosh, Bapi Pradhan, Weihua Lin, Yiyue Zhang, Luca Leoncino, Pavel Chabera, Kaibo Zheng, Eduardo Solano, Johan Hofkens, Tõnu Pullerits

**Affiliations:** † The Division of Chemical Physics and NanoLund, Lund University, Box 124, 22100Lund, Sweden; ∥ Department of Chemistry and Biochemistry, The Ohio State University, 100 West 18th Avenue, Columbus, Ohio43210, United States; § Department of Chemistry, KU Leuven, Celestijnenlaan 200F, 3001Heverlee, Belgium; ¶ Electron Microscopy Facility, Istituto Italiano di Tecnologia, via Morego 30, Genova16163, Italy; δ Max Planck Institute for Polymer Research, Ackermannweg 10, 55128Mainz, Germany; α NCD-SWEET Beamline, ALBA Synchrotron Light Source, Cerdanyola del Vallès, Barcelona, 08290Spain

## Abstract

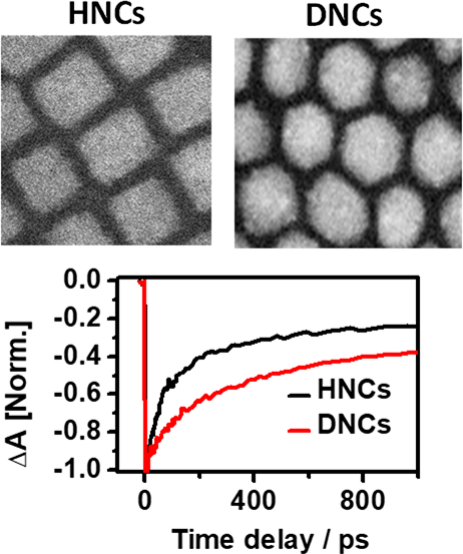

Over the past two decades, intensive research efforts have been devoted to suppressions of Auger recombination in metal-chalcogenide and perovskite nanocrystals (PNCs) for the application of photovoltaics and light emitting devices (LEDs). Here, we have explored dodecahedron cesium lead bromide perovskite nanocrystals (DNCs), which show slower Auger recombination time compared to hexahedron nanocrystals (HNCs). We investigate many-body interactions that are manifested under high excitation flux density in both NCs using ultrafast spectroscopic pump–probe measurements. We demonstrate that the Auger recombination rate due to multiexciton recombinations are lower in DNCs than in HNCs. At low and intermediate excitation density, the majority of carriers recombine through biexcitonic recombination. However, at high excitation density (>10^18^ cm^–3^) a higher number of many-body Auger process dominates over biexcitonic recombination. Compared to HNCs, high PLQY and slower Auger recombinations in DNCs are likely to be significant for the fabrication of highly efficient perovskite-based photonics and LEDs.

Lead halide perovskite nanocrystals (PNCs) have shown exceptional promises in light emitting devices (LEDs) due to their high photoluminescence quantum yield (PLQY), wide tunable emission wavelength range, defect tolerance, and low-cost solution processability.^1−6^ In the past few years, tremendous efforts such as compositional engineering (via doping and alloying),^7,8^ surface reconstruction,^9,10^ shape tuning,^10,11^ and interfacial engineering^12,13^ have been employed to fabricate efficient PNC based LEDs, which led to the significant enhancement in the external quantum efficiencies (EQEs) exceeding 20%.^14^ Even though the EQEs of PNCs are significantly enhanced,^1,15^ still there are numerous complications associated with the device performances and operational stability including defects stemming from Br and Pb vacancies, detachment of ligands from the CsPbBr_3_ surface, leading to a decrease in photoluminescence quantum yield (PLQY), and change of the morphology with time.

Additionally, there are also some limitations in the reported PNC based LEDs due to the severe Auger recombination effect. Due to a lower threshold of carrier density, Auger recombination becomes dominated in PNC.^16,17^ Rapid Auger recombinations is associated with enhanced exciton binding energy (*E*_b_) because of the enhanced Coulomb electron–hole interactions. In practice, Auger recombination is proportional to the third power of E_b_ in strongly confined 1D material.^18^ Accordingly NCs should exhibit strong Auger recombination because of their high *E*_b_.

To overcome the above limitations, PNCs with high PLQY with slow Auger recombination can be a possible solution. Intensive research efforts have been employed to address this issue. Hu et al.^19^ described that Auger recombination slowed down in nonblinking NCs. Recently, Jiang et al.^20^ reported that the Auger recombination can be slowed down by decreasing the dielectric confinement effect of quasi 2D perovskite. It has been noticed that a structural engineering approach is a promising way to suppress the Auger process in NCs and nanowires area.^21−23^

Recently, Zhang et al.^24^ reported a truncated octahedron shape of CsPbBr_3_ with high PLQY using the alkylphosphonate ligand, and they found that new facets are exposed on the surface. Importantly, Pradhan and co-workers synthesized an uncommon noncube morphology including rhombic dodecahedrons and rhombicuboctahedrons of CsPbBr_3_ by changing the reaction conditions.^25,26^ Dodecahedron NCs showed near unity PLQY and retained the high colloidal and phase stability.^27^ The in situ-formed tertiary ammonium ions opened a new facet on the surface, which stabilized the dodecahedron NCs and concurrently reduced the surface defect states. These new nanocrystals would certainly open up more windows for their research in light emitting devices. So far, the explored photophysical properties remain confined to hexahedron NCs (nanocubes and nanoplatelets),^28−31^ while photophysics for these new dodecahedron shaped CsPbBr_3_ NCs are still unknown. Therefore, understanding fundamental photophysical properties of this new type of dodecahedron shaped NCs will be a valuable asset to the researcher for the development of light emitting devices.

In this work, we have studied the transient absorption spectroscopy and kinetics of dodecahedron nanocrystals (DNCs) and compared their dynamics with cube shape hexahedron nanocrystals (HNCs). DNCs showed slower Auger recombination than HNCs, while differences were found on the recombination mechanism depending on the excitation density: at low excitation density, biexciton recombination dominates in both NCs, while at high excitation density, higher order Auger recombination dominates the recombination mechanism.

HNCs and DNCs exhibit a sharp excitonic absorption peak at 509 and 512 nm, respectively, and a red-shifted photoluminescence (PL) maxima at 518 and 522 nm, respectively (Figure 1a,b). To elucidate the structure and composition of HNCs and DNCs, we have used annular dark field-scanning transmission electron microscopy (ADF-STEM) and energy dispersive X-ray spectroscopy (EDS). Figure 1c shows the ADF-STEM image of six faceted hexahedron nanocrystals (HNCs) of CsPbBr_3_. Figure 1d shows ADF-STEM images of 12-faceted rhombic dodecahedron nanocrystals (DNCs) of CsPbBr_3_. The estimated average edge size of both NCs is approximately 12 ± 2 nm. This agrees well with the previously reported CsPbBr_3_ NCs size with this same band gap.^32^ Bandgaps of HNCs and DNCs are calculated from Tauc plot (Figure S1). Figures S2 and S3 show the EDS spectra of HNCs and DNCs. To further confirm the crystal phase, synchrotron grazing incident wide angle X-ray scattering (GIWAXS) experiments were carried out. Figure 1e,f shows the 2D GIWAXS patterns recorded, and Figure S4 shows the corresponding 1D azimuthal profile integration of HNCs and DNCs, where the peak positions match with the standard orthorhombic phase of CsPbBr_3_ (Pbnm),^26^ despite the peak broadening caused by the convolution of the nanocrystalline size of the materials and the grazing incidence geometry employed. The DNCs exhibit the most intense (112) and (020) crystal plane diffraction peaks which retain considerably less intensity in comparison to (110) and (002) for HNCs. Twelve faceted DNCs have two (200), two (020), and eight (112) facets. These 12 facets are completely different from traditional hexahedron NCs which have four (110) and two (002) facets. For HNCs, (110) facets are stabilized with primary ammonium ions (oleylammonium ions), whereas for DNCs (200) and (112) facets are stabilized by tertiary ammonium ions retaining different morphology.^26^ Our pervious DFT calculations reveal a strong dipole moment along the <200> direction,^33^ hence DNCs predominantly consist of polar facets. We have shown the respective planes of the orthorhombic CsPbBr3 crystal structure in the Supporting Information. Moreover, GIWAXS results proved a slight preferential orientation of the nanostructures over the silicon wafer support investigated (several mm^2^), as clearly shown by the brighter arc-shaped sections on the scattering diffraction ring, in agreement with the microscopic STEM, which also showed oriented nanocrystals over the support in real space. Figure 2 panels a and b show transient absorption spectrum (TA) of HNCs and DNCs, respectively, excited at 3.10 eV (400 nm) with an intensity of 2.2 × 10^14^ photons/cm^2^/pulse, corresponding to the initial average generated electron–hole pairs per NC ⟨N⟩ ≈ 31 and average carrier density *n* ≈ 3.8 × 10^18^ cm^–3^ (⟨*N*⟩ = *I*σ, evaluated from the excitation intensity *I* and absorption cross section σ of the NCs).^34^ The average carrier density per NC volume is determined as *n* = ⟨*N*⟩/*V*_NC_, where *V*_NC_ is the NC volume estimated by the average size of the cubic shape NCs.^34^ Both spectra show negative ground state bleach (GSB) signal located around the bandgap, which is due to the state filling effect, and a positive photoinduced absorption (PIA) signal below the bandgap is due to excited-state absorption (ESA) of the photogenerated charges at the excited states. Both NCs with different light intensities show analogous GSB and PIA features, while the time-constants of kinetics vary. If we focus on the key features in the TA spectra on the short time scale (>1 ps), we observe the appearance of a derivative peak shape at the band gap (negative signal on the higher energy side and positive signal at the lower energy side of the band gap). The short-lived derivative peak shape is more obvious at higher excitation intensity, and its dynamics is related to the hot carrier cooling. While HCs relax to the lowest-energy states, the positive PIA signal disappears and is replaced by a strong GSB signal. At the same time, the initial negative GSB increases and reaches the maximum (see Figure 2c and 2d for HNCs and DNCs. respectively).

**Figure 1:**
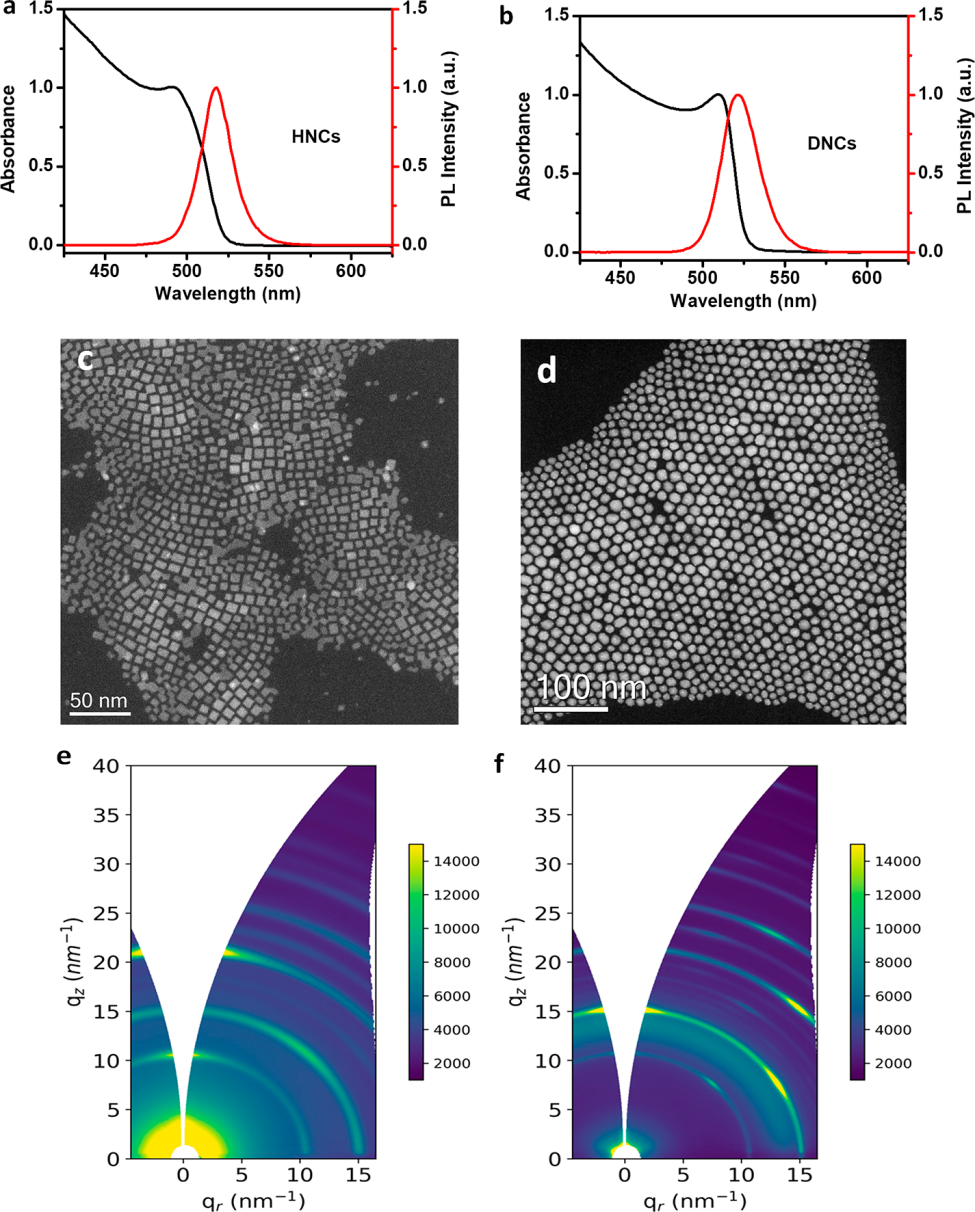
(a) Absorption (black) and PL (red) spectra of HNCs respectively. (b) Absorption (black) and PL (red) spectra of DNCs, respectively. ADF-STEM images of (c) HNCs and (d) DNCs. 2D GIWAXS scattering patterns of (e) HNCs and (f) DNCs.

**Figure 2:**
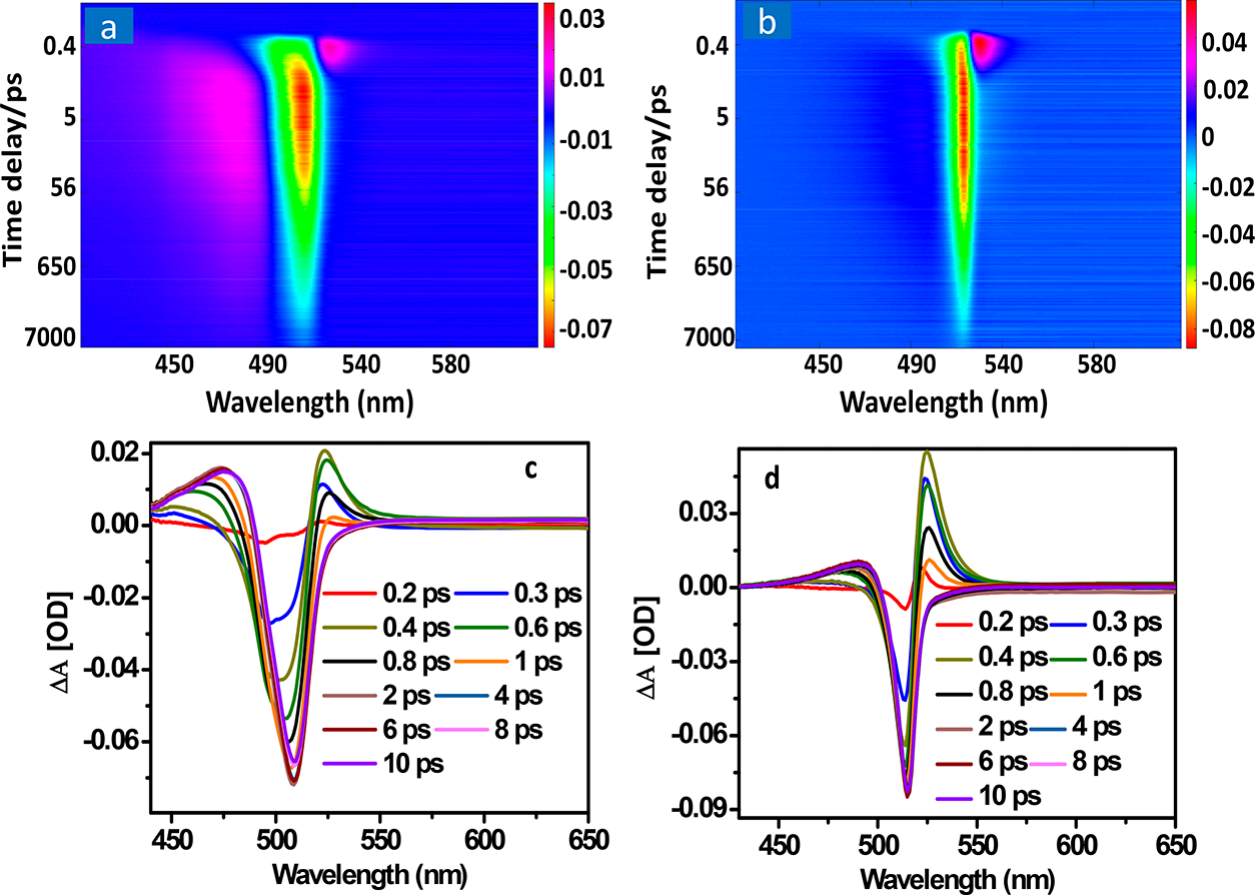
Pseudocolor representation TA spectra of (a) HNCs and (b) DNCs at excitation intensity with ⟨N⟩ ≈ 6.6 (corresponding to *I* = 2.2 × 10^14^ photon/cm^2^/pulse, *n* ≈ 3.8 × 10^18^ cm^–3^). TA spectra showing red shift of the spectra and hot carrier cooling at an early time scale: (c) TA spectra of HNCs and (d) TA spectra of DNCs.

We have calculated the energy shift of the bleach maximum as a function of time delay. It is observed that the shift is maximum at an early time scale, and after 1 ps it is constant (Figure S5). The shift is higher in HNCs (70 meV) than DNCs (10 meV). This also correlates with hot carrier cooling time constants (<1 ps) which is estimated from the exponential fittings of early time kinetics of HNCs (Figure S6b) and DNCs (Figure S7b). The observed red shift of the NCs can be explained by the transient Stark effect.^35−37^ Previously, Aneesh et al.^38^ explained this red shift in early time scale through transient biexcitonic Stark effect in CsPbBr_3_ NCs. For a dipole allowed transition, the shift of transition frequency, , due to an electric field, ε, can be given by^39,40^
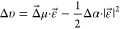
1where  and  are the changes of dipole moment and polarizability between ground and excited states, respectively. The first term in eq 1 signifies the broadening of the spectral shape due to random orientation of Δμ with respect to ε. The second term in eq 1 signifies the shift in the transition energy, with the sign of the shift given by the sign of . The shift of the electronic resonance frequency is correlated with previously reported literature.^41,42^ In general many-body effects lead to time-dependent shifts of the electronic resonance frequency of the NCs due to the dynamics of local fields, which are themselves induced by the presence of excitons.^43^ The local fields induced by the many-body effects resulting from pre-existing electron–hole pairs lead to a shift of the energy of the optical transition.^41^

To analyze the relaxation process of the photogenerated carriers, we have measured the excitation intensity dependent TA decay curves. Figure 3a and 3b display the TA kinetics at different excitation intensity 4.9 × 10^13^ (I1), 1.2 × 10^14^ (I2), 1.6 × 10^14^ (I3), and 2.2 × 10^14^ (I4) photons/cm^2^/pulse for HNCs and DNCs, respectively. I1, I2, I3, and I4 correspond to the average number of excitons per NCs (⟨*N*⟩ ) 1.5, 3.6, 4.8, and 6.6, respectively. TA dynamics of both NCs are monitored at the strong GSB peak (509 and 512 nm for HNCs and DNCs, respectively). Under excitation intensity (*I*), the TA signal of HNCs exhibits a multiexponential decay. The slow component (∼2 ns) indicates the recombination time of a single electron–hole pair, and fast component (∼200 ps) indicates the relaxation time of biexcitonic Auger recombination.^44^ As the excitation intensity increases, the TA signal exhibits an extra fast exponential decay at ∼30 ps. At higher excitation intensity, the TA signal exhibit fast (∼30 ps), intermediate (∼200 ps), and long (∼2 ns) decay component. Similarly DNCs also show two components at low excitation intensity, a slow component appears ∼4 ns and a fast component appears around 350 ps. However, at high excitation intensity DNCs also exhibit a very fast component ∼2 ps. The origin of the intermediate and fast decay is the Auger recombination due to low and high number of exciton–exciton interactions, and slow component is due to single exciton recombinations.^44,45^ It is important to mention that photochemical doping caused the ionization of the NCs and subsequently formed trion in CsPbBr_3_ perovskite NCs. Trion recombination is a competitive process with biexciton recombination.^46^ In the case of photochemical doping, a reducing agent is used to extract the photogenerated hole from the NC, leaving behind an excess electron to the CB of the NC. However, in our case we exclude that possibility as we did not use any reducing agent as photochemical doping in our samples. Nakahara et al.^47^ reported that trion formation in CsPbBr3 perovskite is due the presence of surface traps. To prove the relation between surface traps and trion formation they treated the sample with surface passivating agents’ sodium thiocyanates (NaSCN) and found the improved PLQY. Further, they conducted TA measurements for the surface treated samples with static and stir conditions and observed almost similar lifetimes as those of the untreated samples. Therefore, we consider that carrier recombination is going through monoexciton, biexciton, and higher number of many-body recombination process. Trinh et al.^42^ describe both two-body and many-body recombination in methylammonium lead iodide (MAPbI_3_) perovskites. To elucidate, the recombination kinetics at the band edge of both NCs over a range of excitation intensities can be modeled by the simple rate equations. When the excitation intensity is low, the major decay routes for band edge excitons are mono- and biexciton recombinations. If the Auger process occurs due to biexciton recombinations then the rate equation can be described as^42,48^
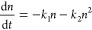
2where *n* is the exciton density per NCs, *k*_1_ is the first-order rate constant corresponding to single exciton recombination, and *k*_2_ is the second-order rate constant for biexciton recombination. Equation 2 can be solved to yield^49^
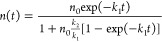
3where *n*_0_ is the initial exciton density. The kinetics corresponding to I1 was fitted with eq 3 assuming *k*_1_ = 3.3 × 10^8^ s^–1^ and 2.8 × 10^8^ s^–1^ for HNCs and DNCs, respectively, which was obtained from long component of TA kinetics. The value of *k*_2_ is estimated from the fitting and found to be 2.8 × 10^–11^ cm^3^ s^–1^ and 1.5 × 10^–11^ cm^3^ s^–1^ for HNCs and DNCs, respectively. Figure 3 panels c and d show the fitting results of HNCs and DNCs, respectively, with eq 3. From both panels it can be observed that at lower intensity, it is fitted well with eq 3, but at higher intensity experimental data are not in good agreement with the fitted curves.

**Figure 3:**
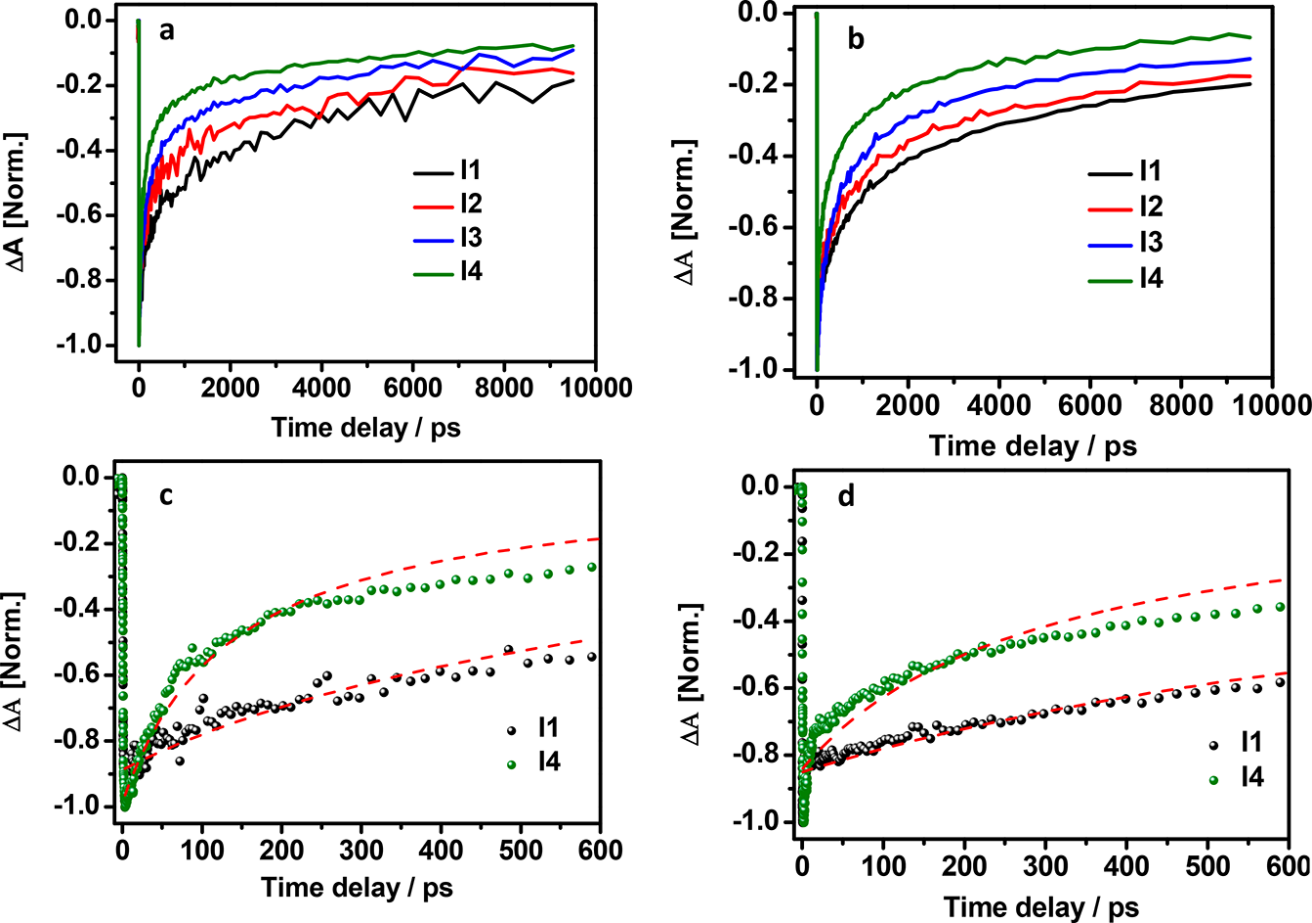
Transient absorption (TA) kinetics of (a) HNCs and (b) DNCs at different excitation intensities: I1 = 4.9 × 10^13^, I2 = 1.2 × 10^14^, I3 = 1.6 × 10^14^, and I4 = 2.2 × 10^14^ photon/cm^2^/pulse. (c) TA kinetics of HNCs and (d) TA kinetics of DNCs at excitation intensities I1 (black) and I4 (olive); dashed red lines are the fitted curves based on eq 2. Excitation wavelength was 400 nm, and detection wavelength was 509 and 512 nm for HNCs and DNCs, respectively.

From the above exciton–excition interaction model, we can observe that biexcitonic recombination is slower in DNCs than in HNCs. We also observe from multiexponential fittings (Figures S6 and S7) that DNCs show an intermediate component characterized by a time constant on the order of hundreds of picoseconds after excitation at 400 nm, having much-reduced amplitude compared to the HNCs (Tables S3 and S4) which leads to a reduction of the Auger induced nonradiative pathways at high excitation exposition. Figure 4 panels a–d describe the TA kinetics of both HNCs and DNCs at different excitation intensities I1, I2, I3, and I4. Figure 4, clearly shows slower Auger recombination kinetics of DNCs compared to HNCs.

**Figure 4:**
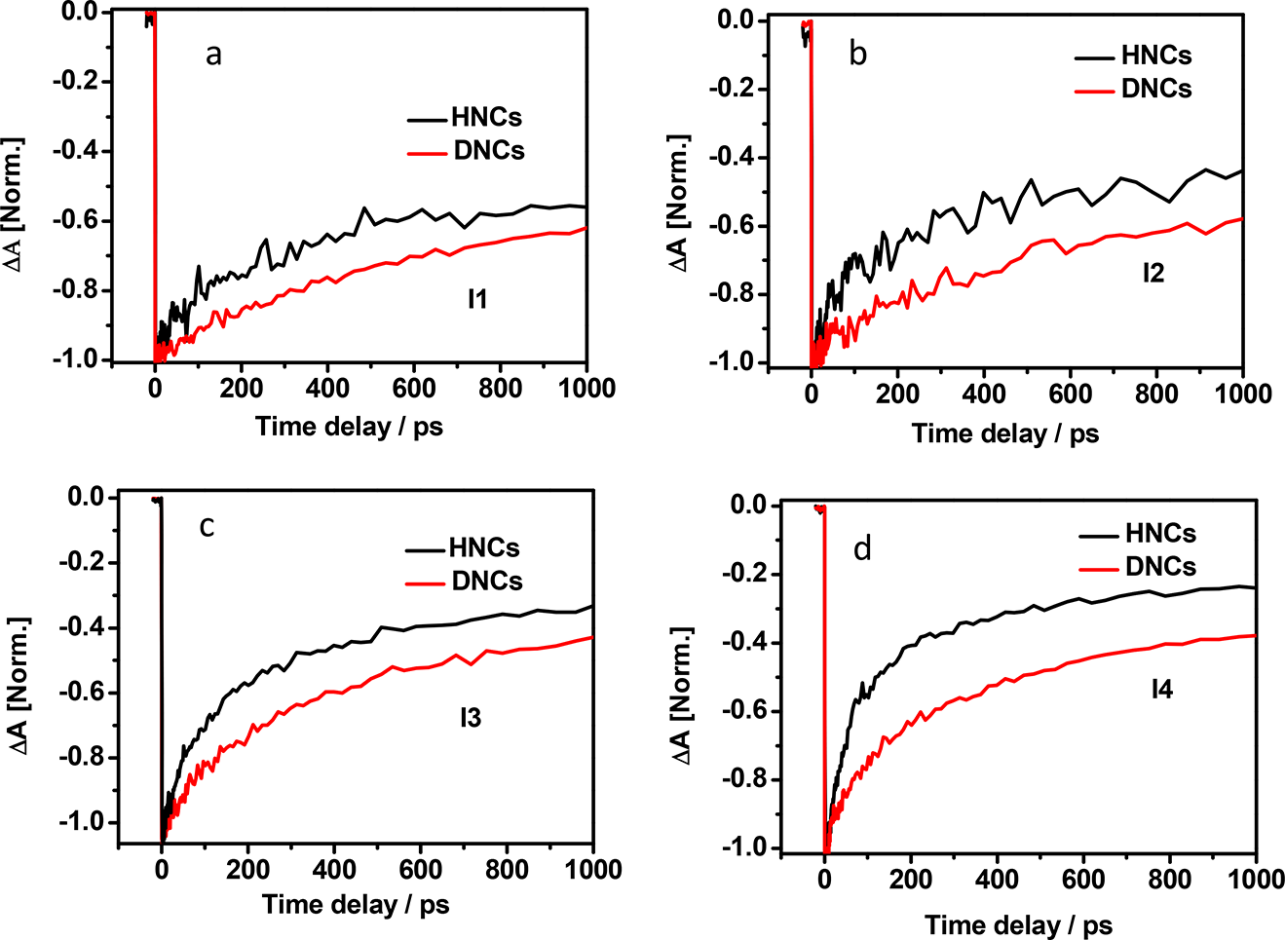
Comparison of TA kinetics of HNCs and DNCs at excitation intensity (a) I1, (b) I2, (c) I3, and (d) I4.

Faster Auger recombination is an important challenge faced by PNCs, which causes significant efficiency roll-off and impedes their further commercialization. As the Auger recombination rate is proportional to the exciton binding energy (*E*_b_); thereby, the Auger process might be slowed down by reducing the corresponding *E*_b_. Dielectric constant may influence the *E*_b_ of the DNCs.^42^ According to a previous manuscript,^50^ DNCs contain an extra new polar facet, it affects the magnitude of polarization achievable, and hence the dielectric constant. Therefore, DNCs contain a higher polarity than HNCs which consequently reduces the *E*_b_. In practice, the Auger recombination rate is proportional to the third power of the *E*_b_ in strongly confined systems.^18^ Accordingly, DNCs should exhibit slower Auger recombination than HNCs. In 2D perovskite based light emitting devices, slower Auger recombination reduces the Joule heating and enhance the device stability under high current density.^20^ Therefore, this work may open a new opportunity for the further development of PNC based high efficient photonic devices in the near future.

In summary, we have studied the many body exciton recombination dynamics in HNCs and DNCs at different excitation regimes using femtosecond transient absorption spectroscopy. We have demonstrated slower Auger recombination in DNCs than in HNCs. We attribute that the slow Auger recombination is due to lower exciton binding energy of DNCs than that of HNCs. Furthermore, our results reveal that DNCs possess lower transient Stark effect compared to HNCs. The generation of hot carriers in NCs causes a transient Stark effect leading to the spectral red-shift at early time scale. In both perovskites, excitonic and biexcitonic recombinations are major decay routes. Auger recombination due to higher number of many body exciton recombination is significant at very high excitation densities (>10^18^ cm^–3^). Our results show that DNCs can be more suitable for photonics and LEDs applications.

## Supporting Information Available

Material characterizations, time dependent energy shift calculations, and multiexponential fitting results of HNCs and DNCs of CsPbBr_3_ perovskites (PDF)
