# Supplementary material for: Slower Auger Recombination in 12-Faceted Dodecahedron CsPbBr3 Nanocrystals
Source: J Phys Chem Lett. 2023 Jan 25;14(4):1066–72. doi: 10.1021/acs.jpclett.2c03389 (PMC10334461; doi:10.1021/acs.jpclett.2c03389)
Supplement: Supplementary file 1 — jz2c03389_si_001.pdf [file jz2c03389_si_001.pdf]

Supporting Information

Slower Auger Recombination in 12-Faceted Dodecahedron $CsPbBr_3$ Nanocrystals

Supriya Ghosh[†,‖#*], Bapi Pradhan[§#], Weihua Lin[†], Yiyue Zhang[§], Luca Leoncino[¶], Pavel Chabera[†], Kaibo Zheng[†], Eduardo Solano[α], Johan Hofkens[§, δ] and Tõnu Pullerits[†*]

[†]The Division of Chemical Physics and NanoLund, Lund University, Box 124, 22100 Lund, Sweden

[‖]Department of Chemistry and Biochemistry, The Ohio State University, 100 West 18th Avenue, Columbus, Ohio 43210, USA

[§]Department of Chemistry, KU Leuven, Celestijnenlaan 200F, 3001 Heverlee, Belgium

[¶]Electron Microscopy Facility, Istituto Italiano di Tecnologia, via Morego 30, Genova 16163, Italy

[δ]Max Planck Institute for Polymer Research, Ackermannweg 10, 55128 Mainz, Germany

[α]NCD-SWEET Beamline, ALBA Synchrotron Light Source, Cerdanyola del Vallès, Barcelona, 08290 Spain

AUTHOR INFORMATION

[#]These authors contribute equally

Corresponding Authors

* Tonu.Pullerits@chemphys.lu.se +46 46 222 81 31

* ghosh.270@osu.edu +1 614 360 4134

Experimental Sections

Synthesis of cesium oleate. In a typical synthesis,[1] 0.4 g of Cs_2CO_3 was loaded in a 100 ml three-neck round-bottom flask along with 15 mL 1-octadecene (ODE) and 1.725 mL oleic acid (OA). The reaction mixture was degassed under vacuum for 1 h at 120 °C, then the reaction temperature was set to 150 °C for 10 min to ensure the formation of a clear Cs-oleate solution. The solution was then collected under hot conditions and stored in a nitrogen filled vial. After cooling to the room temperature, the mixture was solidified. Thus, cesium oleate needs to be heated to liquefy above 80 °C before further use.

Synthesis of dodecahedron $CsPbBr_3$ NCs. In a typical synthesis method[2], 89.2 mg of lead oxide, 238.8 mg of phenacyl bromide, 2 mL OA, and 10 mL ODE were loaded in a 50 mL three-neck round-bottom flask. Then, the mixture was evacuated and filled with nitrogen under stirring at room temperature. After the above steps were repeated three times, the whole system was degassed with nitrogen for 1 h at 120 ° C. After all the salts were dissolved, the reaction temperature was increased, and 1 mL oleylamine (OLAM) was injected at 220 °C. The solution became red initially within 1 min, and then gradually turned to yellow around 20 min. After that, 0.8 mL of previously prepared Cs-oleate was swiftly injected into the clean yellow solution at 180 °C temperatures and annealed for 15 min, to form DNCs with different particle sizes. Once reaching the reserved time, samples were collected with ice quenching. For purifying the final PNCs, crude samples and 30 mL methyal acetate (MeOAc) were taken in a 50 ml centrifuge tube and centrifuged at 8700 rpm for 10 min. After centrifugation, the supernatant solution was discarded, and the precipitate was redispersed in 4 mL hexane for further use.

Synthesis of hexahedron $CsPbBr_3$ NCs. As for the synthesis of hexahedron $CsPbBr_3$ NCs, we used a modified typical method developed from Kovalenko et. al.[1] and Kuno et. al.[3] Specifically, 266 mg lead bromide, 2 mL OA, 2mL OLAM and 10 mL ODE were loaded in a

50 mL three-neck round-bottom flask and degassed under vacuum for 1 h at 120 ° C. Then the temperature was increased to 220 °C and 1.6 mL of Cs-oleate was swiftly injected. Samples were collected with ice quenching immediately within 10s. The same purify method as hexagonal NCs was used to collect the final cube NCs. The precipitate was redispersed in 4 mL hexane for further use.

Synchrotron-based Grazing Incidence Wide Angle X-ray Scattering (GIWAXS). Data were recorded at NCD-SWEET beamline (ALBA synchrotron in Cerdanyola del Vallès, Spain) with a 12.4 KeV (λ = 0.9998 Å) monochromatic X-ray beam of 150 × 100 μm^2 [H × V], which was prepared using a Si (111) channel cut monochromator and an array of Be collimating lenses. The scattered signal was recorded using a Rayonix LX255-HS area detector (88.54 × 88.54 μm^2 [H × V] pixel size for the image binning set of 2 × 2) placed at 242.334 mm from the sample position. The reciprocal q-space and sample-to-detector distance were calculated using Cr_2O_3 as calibrant. An incident angle (α_i) of 0.5° was chosen to ensure full penetration of the X-ray beam through the layer. Continuous N_2 flow over the sample was employed during the measurements. Collected 2D images were azimuthally integrated using PyFAI.[4]

Transient Absorption (TA) Measurements

TA experiments were performed by using a femtosecond pump-probe setup. Briefly**,** The broadband femtosecond pump probe measurements were carried out based on a Solstice (Spectra Physics) amplified laser system that produces ~60 fs pulses at a central wavelength of 796 nm at 4 kHz repetition rate. The laser output is split into two parts to generate pump and probe beams. The pump pulses were produced by a collinear optical parametric amplifier (TOPAS-C, Light Conversion). A second TOPAS was used to generate 1350 nm pulses,

which were focused on a CaF2 crystal to generate broadband white light probe. The mutual polarization between pump and probe beams was set to the magic angle (54.7°) by placing a Berek compensator in the pump beam. The pump and probe pulses were made overlapped at the sample position. To eliminate low frequency laser noises, probe was splitted into two beams and detected as sample and reference separately. The detection of probe pulses was performed under pump blocked and unblocked conditions with a mechanical chopper of rotational frequency 500 Hz. TA spectra were recorded by CCD arrays using a grating spectrograph. Correction for the group velocity dispersion (GVD) of WLC spectra was carried out at the data processing stage.

Steady-State Spectroscopy

Ground-state absorption spectra were measured in an UV-vis absorption spectrophotometer (PerkinElmer, Lambda 1050) equipped with an integrated sphere to exclude signal due to light scattering. Steady-state photoluminescence was measured using a standard spectrometer (Horiba, Spex 1681) with excitation at 400 nm.

Photoluminescence quantum yields (PLQY) were recorded on a Horiba spectrofluorimeter with samples placed in a quartz cuvette (1 mm) path length. The excitation wavelength was 395 nm with an emission window 420-600 nm. The estimated PLQY for HNCs is 47.3±5.4% and DNCs 70.2±5.8% respectively.

Transmission Electron Microscopy

Transmission Electron Microscopy (TEM) specimens were prepared by drop-casting a suspension of $CsPbBr_3$ NCs in toluene onto carbon-coated copper grids. Annular Dark Field Scanning TEM (ADF-STEM) images have been obtained by Tecnai G2 F20 TWIN TMP microscope (Schottky emitter), operated at 200 kV (Fig. 1c), and by a JEOL JEM-1400Plus

equipped with thermionic source (LaB_6, operated at 120 kV) and with a silicon drift type EDS detector JEOL Dry SD30GV (Fig. 1d). Statistics on the edge size of NCs were done on ADF-STEM images of the two types of NCs (around 100 NCs) using Gatan Digital Micrograph software.

Tauc plot

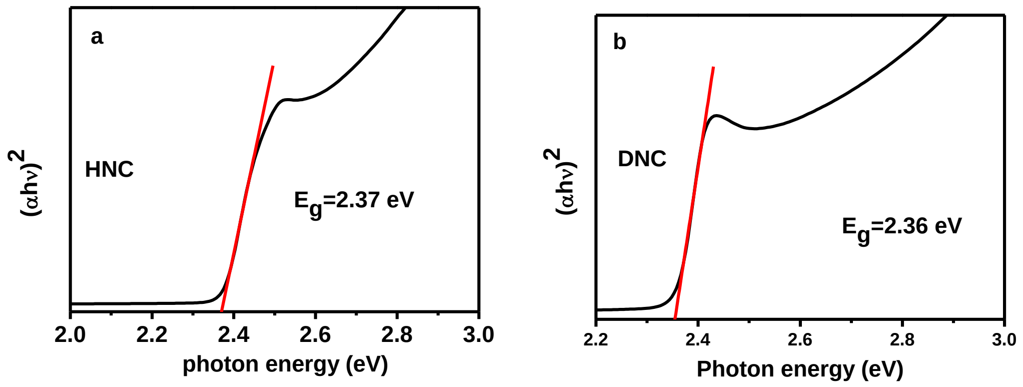

Figure S1. Tauc plot of (a) HNC and (b) DNC respectively.

EDS spectrum of HNCs

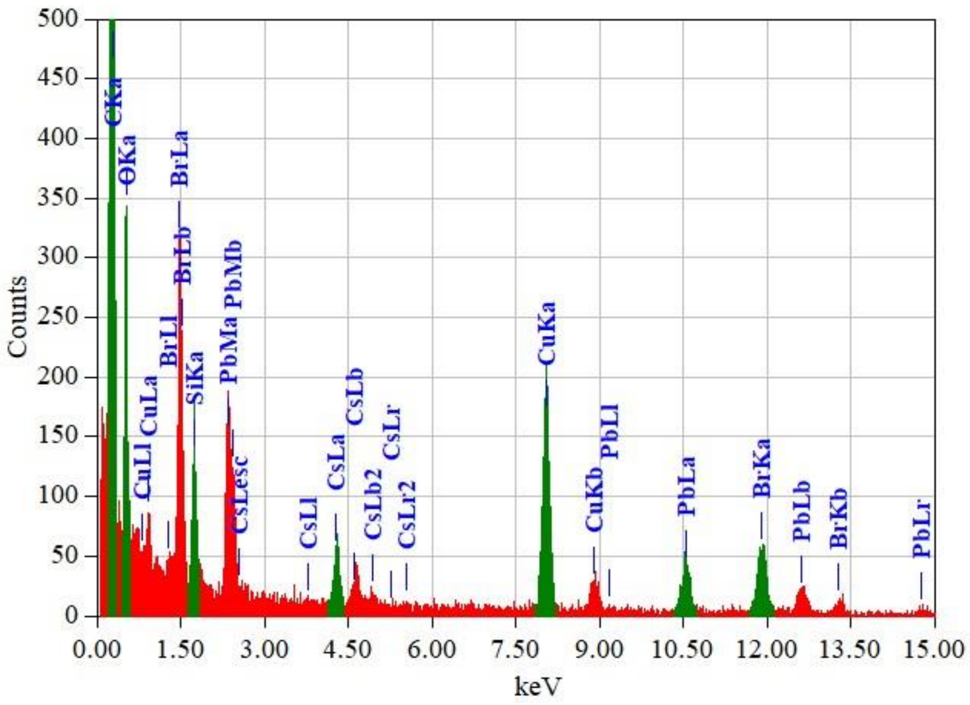

Figure S2. EDS spectrum acquired on the overall area of 100 nm^2. Cs, Pb and Br are clearly detected and quantified in the table below. Cu and C signals are due to TEM grid, unexpected presence of Si may due to TEM grid manufacturing process.

Table S1. Fractional contributions of different atoms in HNCs

Element		(keV)	Counts	Mass%	Atom%
C K	Excluded			ND	ND
O K	Excluded			ND	ND
Si K	Excluded			ND	ND
Cu K	Excluded			ND	ND
Br K	Ref.	11.907	1053.9	38.61	56.94
Cs L		4.286	671.11	25.63	22.73
Pb L		10.55	663.11	35.76	20.34
Total				100	100

EDS spectrum of DNCs

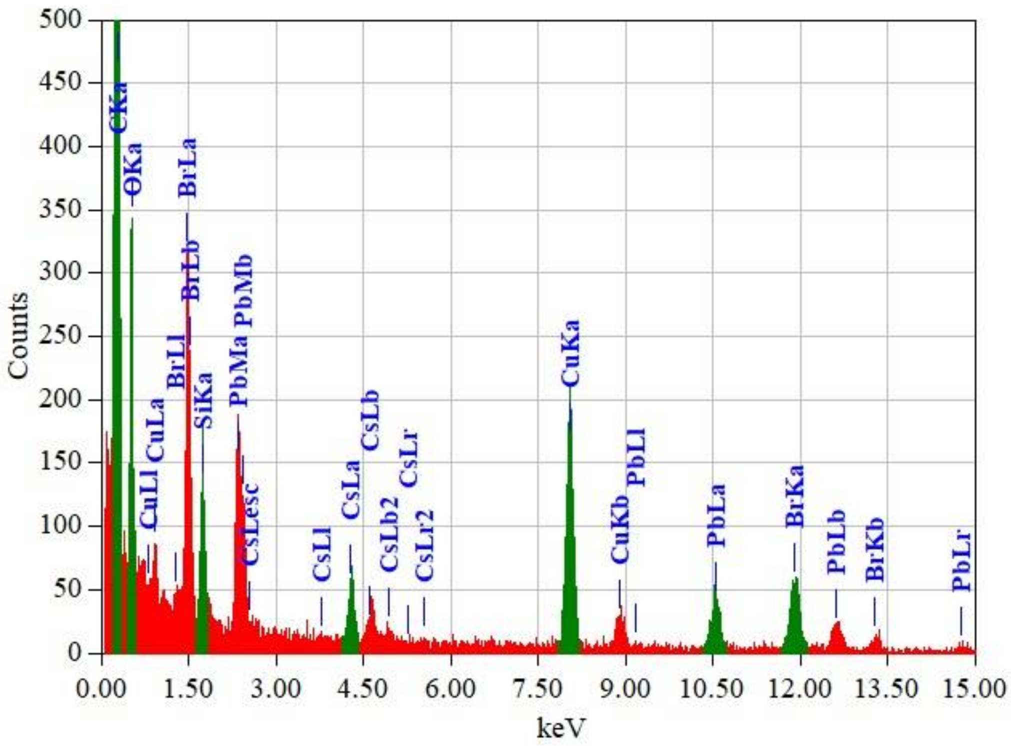

Figure S3. EDS spectrum acquired on the overall area, Cs, Pb and Br are clearly detected and quantified in the table below. Cu and C signals are due to TEM grid.

Table S2. Fractional contributions of different atoms in HNCs

Element		(keV)	Counts	Mass%	Atom%
C K	Excluded			ND	ND
O K	Excluded			ND	ND
Cu K	Excluded			ND	ND
Br K	Ref.	11.907	4319.7	38.82	56.38
Cs L		4.286	3188.07	29.87	26.08
Pb L		10.55	2366.4	31.3	17.53
Total				100	100

X-ray defraction (XRD) patterns of HNCs and DNCs

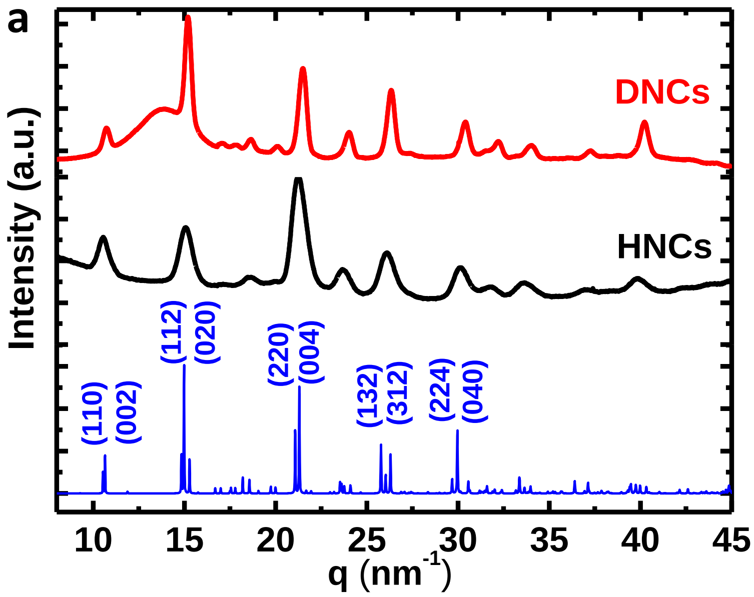

Figure S4. (a) 1D azimuthally integrated profiles of HNCs (black) and DNCs (red) respectively, which match (blue) with orthorhombic $CsPbBr_3$ phase (ICSD#14608).

Estimated red shift of bleach maximum of HNCs and DNCs respectively

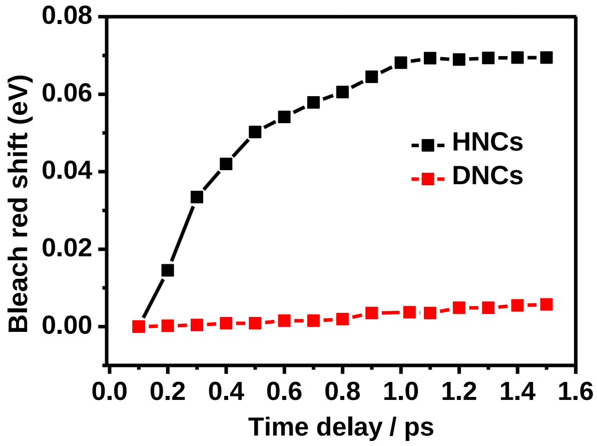

Figure S5. Redshift of bleach band as a function of time delay. Black and red squares represent the red shift of bleach band as a function of time delay in HNCs and DNCs respectively.

Multiexponential fiiting of HNCs

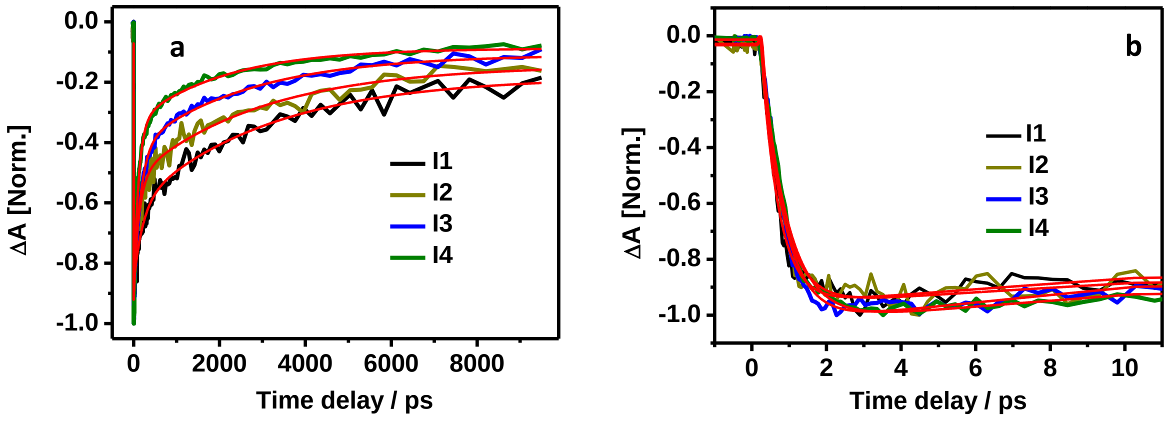

Figure S6. Multiexponential fittings of TA kinetics of HNCs at different excitation intensities: I1 (black), I2 (dark yellow), I3 (blue) and I4 (olive) (a) at longer scale and (b) at shorter scale (<10 ps). Excitation wavelength was 400 nm and detection wavelength was 509 nm and 512 nm for HNCs and DNCs respectively.

Multiexponential fiiting of DNCs

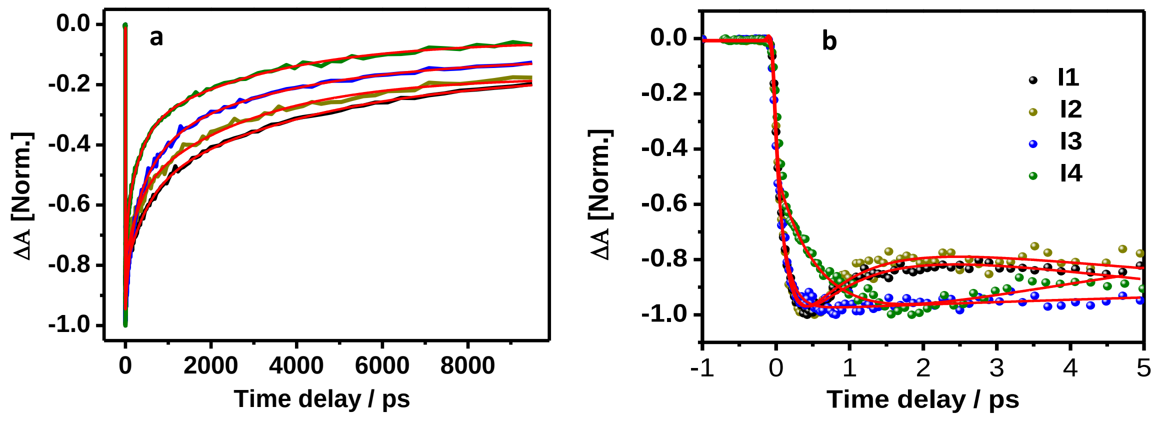

Figure S7. Multiexponential fittings of TA kinetics of DNCs at different excitation intensities: I1 (black), I2 (dark yellow), I3 (blue) and I4 (olive) (a) at longer scale and (b) at shorter scale (<10 ps).

Fitting Parameters

Table S3. Fitting parameters determined for TA kinetics of HNCs

**Sample**	**τ_1 (ns)**	**τ_2 (ns)**	**τ_3 (ns)**
I1	52 (17%)	511 (31%)	3002 (52%)
I2	33 (29%)	292 (30%)	3044 (41%)
I3	27 (43%)	250 (27%)	3011 (30%)
I4	22 (60%)	215 (21%)	3000 (19%)

Table S4. Fitting parameters determined for TA kinetics of DNCs

Sample	τ_1 (ns)	τ_2 (ns)	τ_3 (ns)
I1	1.89<br>(14%)	515<br>(33%)	4137<br>(53%)
I2	1.45<br>(21%)	329<br>(28%)	2810<br>(51%)
I3	2.7<br>(31%)	250<br>(27%)	3011<br>(42%)
I4	1.48<br>(47%)	304<br>(22%)	3081<br>(31%)

Different crystal planes

200 plane

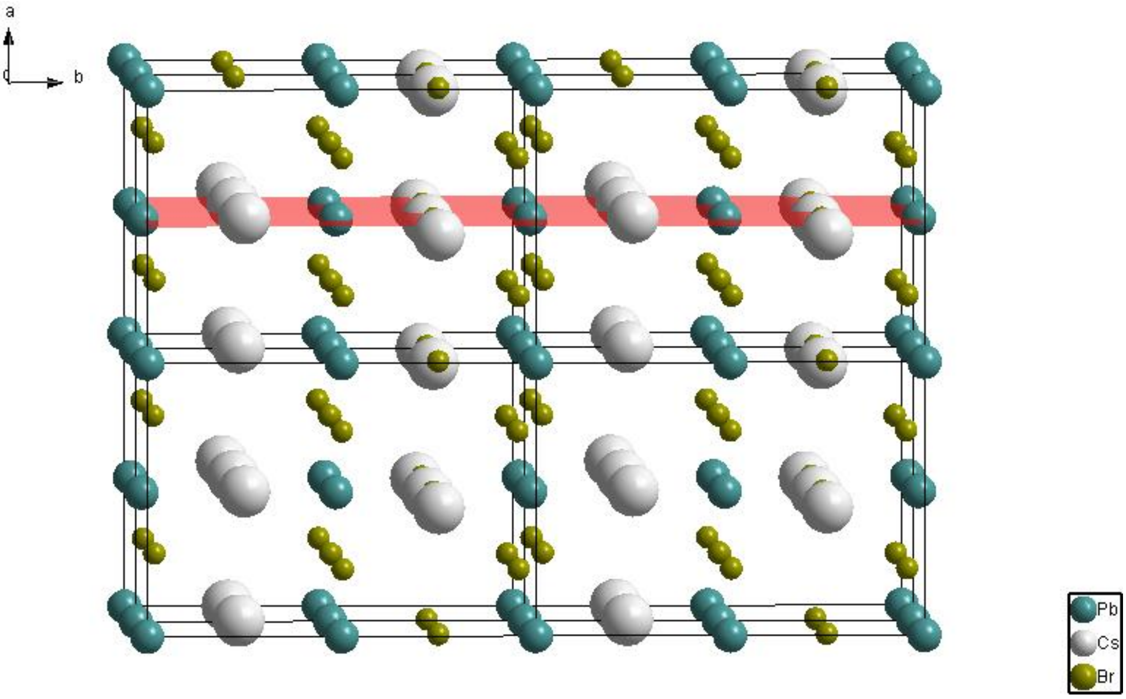

110 plane

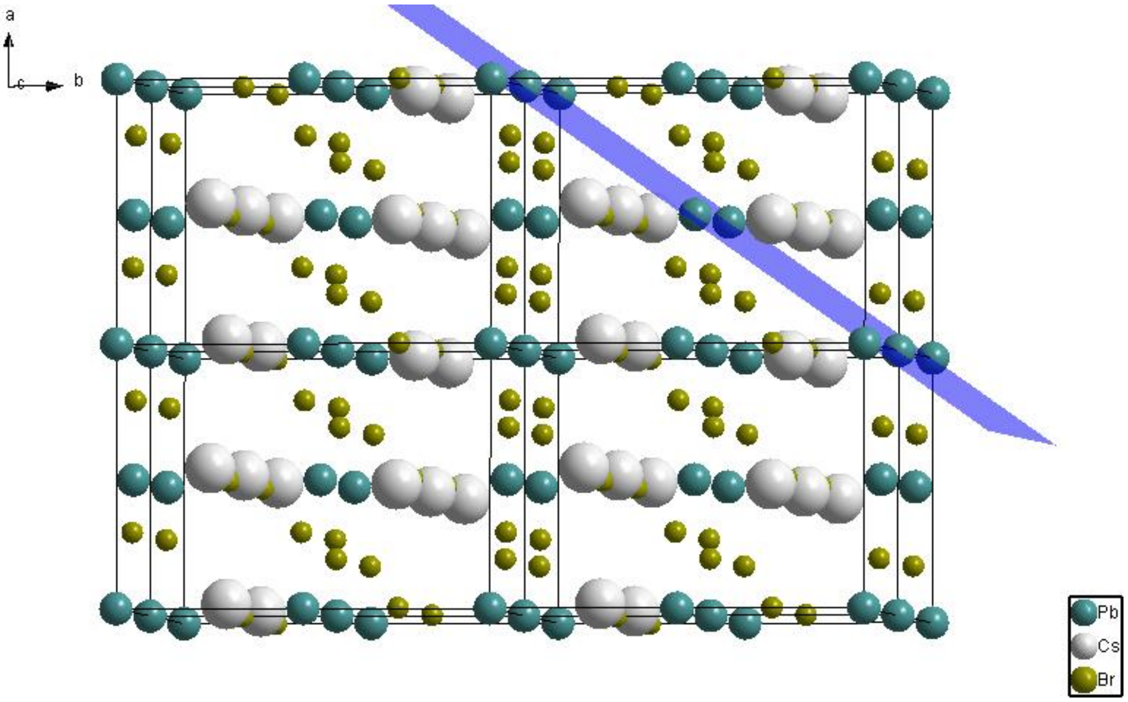

112 plane

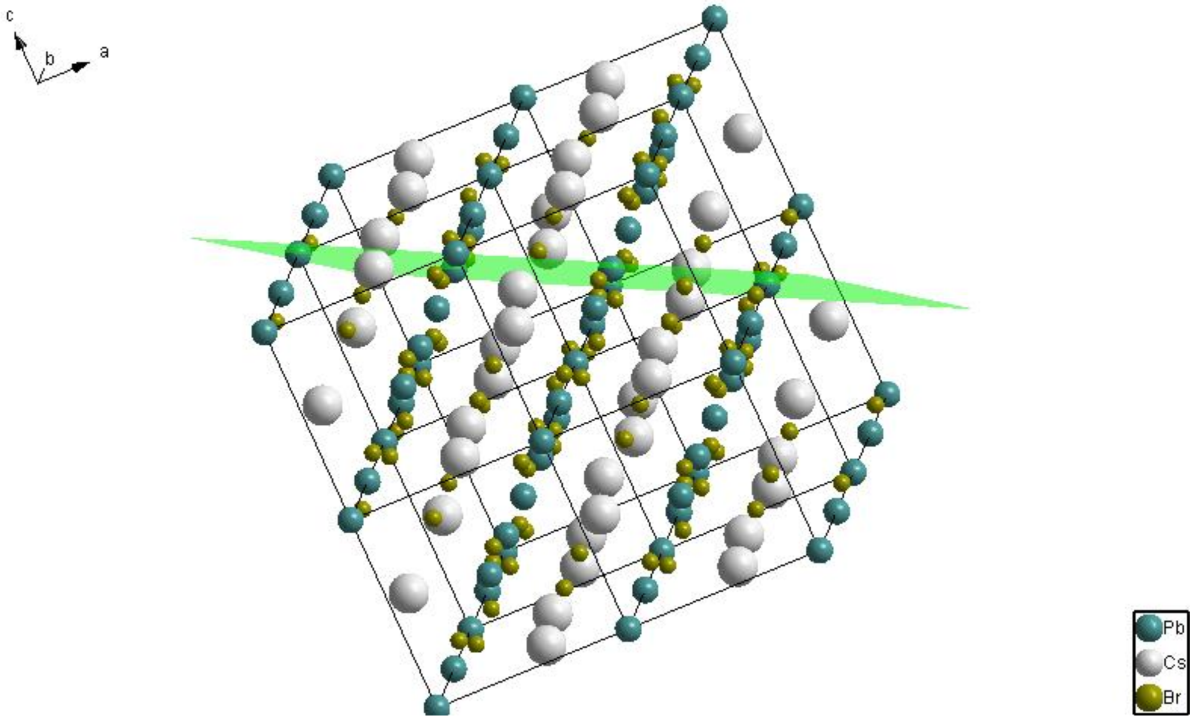

200, 110 and 112 planes together

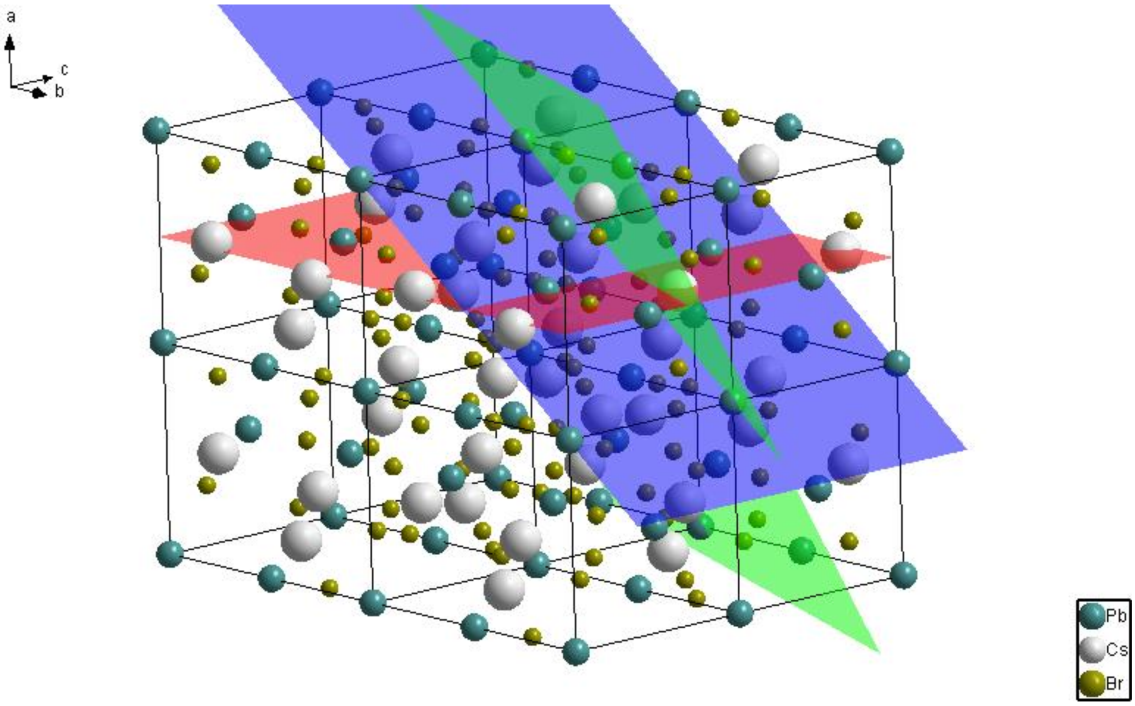

References

1. Protesescu, L.; Yakunin, S.; Bodnarchuk, M. I.; Krieg, F.; Caputo, R.; Hendon, C. H.; Yang, R. X.; Walsh, A.; Kovalenko, M. V. Nanocrystals of Cesium Lead Halide Perovskites (Cspbx3, X= Cl, Br, and I): Novel Optoelectronic Materials Showing Bright Emission with Wide Color Gamut. *Nano Lett.* **2015**, *15*, 3692-3696.

2. Bera, S.; Behera, R. K.; Pradhan, N. A-Halo Ketone for Polyhedral Perovskite Nanocrystals: Evolutions, Shape Conversions, Ligand Chemistry, and Self-Assembly. *J. Am. Chem. Soc.* **2020**, *142*, 20865-20874.

3. Brennan, M. C.; Herr, J. E.; Nguyen-Beck, T. S.; Zinna, J.; Draguta, S.; Rouvimov, S.; Parkhill, J.; Kuno, M. *J. Am. Chem. Soc.* **2017**, *139*, 12201

4. Kieffer, J.; Karkoulis, D. PyFAI, a Versatile Library for Azimuthal Regrouping. *J. Phys.: Conf. Ser.* **2013**, *425*, 202012– 2012017
